# SLC25A21 correlates with the prognosis of adult acute myeloid leukemia through inhibiting the growth of leukemia cells via downregulating CXCL8

**DOI:** 10.1038/s41419-024-07308-y

**Published:** 2024-12-20

**Authors:** Yu Liu, Yan Xu, Qianqian Hao, Luyao Shi, Yufei Chen, Yajun Liu, Mengya Li, Yu Zhang, Tao Li, Yafei Li, Zhongxing Jiang, Yanfang Liu, Chong Wang, Zhilei Bian, Lu Yang, Shujuan Wang

**Affiliations:** 1 Department of Hematology, The First Affiliated Hospital of Zhengzhou University, Zhengzhou, China; 2 Department of Orthopaedics, Brown University, Warren Alpert Medical School/Rhode Island Hospital, Providence, RI USA

**Keywords:** Acute myeloid leukaemia, Prognostic markers

## Abstract

In recent years, targeting mitochondrial apoptosis has emerged as a promising therapeutic strategy for Acute Myeloid Leukemia (AML). The SLC25 family of mitochondrial carriers plays a critical role in maintaining mitochondrial function and regulating apoptosis. However, the role of *SLC25A21*, an oxodicarboxylate carrier, in AML progression and its potential as a prognostic biomarker remain underexplored. This study aimed to further investigate the role, molecular mechanism, and potential clinical value of *SLC25A21* in AML progression. The transcript levels of *SLC25A21* in bone marrow specimens were analyzed using real-time quantitative polymerase chain reaction. The correlation between *SLC25A21* expression and the prognosis of AML was assessed through survival analysis. Findings revealed that *SLC25A21* was downregulated in adult AML, and the low expression of *SLC25A21* was correlated with worse prognosis for AML patients. Furthermore, overexpression of *SLC25A21* inhibited cell proliferation and cell cycle progression, and was correlated with apoptosis through mitochondrial apoptosis signaling pathway. C-X-C motif chemokine ligand 8 (*CXCL8*) was identified as a downstream target of *SLC25A21*. These functions of *SLC25A21* could be rescued by the overexpression of *CXCL8*. Moreover, *SLC25A21* overexpression significantly suppressed the growth of xenograft tumors. In conclusion, the low *SLC25A21* expression is correlated with poor clinical outcome. The overexpression of *SLC25A21* inhibited the AML cell survival and proliferation by dysregulating the expression of *CXCL8*. *SLC25A21* might be a potential prognostic marker and a treatment target for AML.

## Introduction

Acute myeloid leukemia (AML) is a highly heterogeneous bone marrow stem cell cancer characterized by a broad spectrum of molecular mutations and genomic changes. AML is the most common form of acute leukemia in adults [1]. The 5-year overall survival rate is 50% in young AML patients, but is less than 10% in older AML patients with age over 60 [2]. The updated 2017 European LeukemiaNet (ELN) risk stratification guidelines include cytogenetic abnormalities and genetic mutations, which have been widely used to guide the treatment and/or predict the outcomes of AML patients [3].

The application of targeted therapies, including targeting fms-like tyrosine kinase 3 (*FLT3*), and CAR-T cell treatments, has significantly improved the prognosis of AML patients [4, 5]. Mitochondrial dysfunction plays a critical role in AML pathogenesis, influencing the metabolic characteristics of leukemia stem cells (LSCs) and AML blasts [6]. Targeted therapies focusing on mitochondrial pathways, such as the BCL-2 inhibitor Venetoclax, show promise in eradicating LSCs and preventing AML relapse [7, 8]. The growing understanding of the interplay between mitochondrial dysfunction and apoptotic pathways provides a promising avenue for the development of targeted treatments in AML [6, 9]. Even with all these developments, challenges with relapse, mortality, primary and secondary treatment resistance persist, underscoring the need for new molecular targets and personalized therapies.

The Solute Carrier Family 25 (SLC25) family is the largest group of the solute carrier superfamilies, comprising 53 human genes involved in various metabolic pathways and cellular functions [10]. Solute carrier 25 member 21 (*SLC25A21*), also known as oxodicarboxylate carrier (ODC), is located on chromosome 14q13.3. The deficiency of germline *SLC25A21* in human leads to the depletion of mitochondrial DNA and results in a disease resembling spinal muscular atrophy [11]. Studies suggested that *SLC25A21* was associated with the prognosis of glioma [12], bladder cancer (BCa) [13] and colorectal cancer (CRC) [14]. However, the role of *SLC25A21* in AML is still unclear.

*SLC25A21* is responsible for the transport of 2-oxoadipate and α-ketoglutarate (α-KG) across the inner mitochondrial membrane [15]. Overexpression of *SLC25A21* results in the efflux of mitochondrial α-KG [14]. The lactate-induced C-X-C motif chemokine ligand 8 (*CXCL8*) expression could be suppressed by α-KG [16]. Thus, *CXCL8* may be involved in the mechanism of *SLC25A21* regulating the biological function of AML. *CXCL8*/interleukin 8 (IL8), a member of the CXC chemokine family, could promote tumor invasion, migration, angiogenesis, and metastasis by binding to its receptors CXCR1 and CXCR2 [17, 18]. It enhances the fitness of leukemic stem cells in various hematologic malignancies, including AML [19, 20], and may link mesenchymal stromal cells and hypoxia in AML [21]. The overactivation of the IL8 pathway increases the proliferation and viability of leukemia stem cells, impacting the stromal microenvironment [22].

Our study indicated that low *SLC25A21* expression was associated with poor prognosis in AML patients. Aberrant expression of *SLC25A21* influenced AML cell biological behavior of both in vitro and in vivo, inhibiting cell proliferation, inducing G0/G1 arrest, and promoting apoptosis. Rescue experiments showed that overexpression of *CXCL8* could reverse these functions. These results demonstrated that *SLC25A21* could modulate AML cell functionality through repressing*CXCL8*.

## Methods

Additional methods can be found in Supplementary Materials and Methods.

### Subjects

Bone marrow samples were obtained from 236 patients newly diagnosed with AML and 22 healthy donors at the First Affiliated Hospital of Zhengzhou University between February 2017 and March 2020. WHO-classified AML and treatment with at least one course were the inclusion criteria. The exclusion criteria were patients who did not receive treatment or received treatment in other hospitals and patients with acute promyelocytic leukemia. Clinical information and therapy regime have been published [23]. Subjects were followed up until death, loss to follow-up or December 2023. The diagnosis of AML, complete remission (CR), relapse, risk stratification and overall survival (OS) were defined according to NCCN guideline for acute myeloid leukemia Version 2.2021 [24]. The study was approved by the Ethics Committee of the First Affiliated Hospital of Zhengzhou University. Informed consent was obtained from all patients according to the Declaration of Helsinki.

### Lentiviral transduction

For *SLC25A21* overexpression, Kasumi-1 and THP-1 cells were stably transfected with *SLC25A21* overexpression lentivirus (Genechem, Shanghai, China) or control lentiviral (Genechem). Cells were infected at a multiplicity of infection (MOI) of 100 for 12 h. After 72 h of transduction, culture media was supplemented with 2 μg/ml puromycin dihydrochloride (Genechem) to select stably transfected cells. Then, to induce *SLC25A21* overexpression, 6 μg/mL doxycycline (MCE, NY, USA) was added to the medium for 3 days. The transfection efficiency was confirmed by RT-qPCR and western blot. Cell transfection and *CXCL8* overexpression were performed as previously described [25].

### RNA extraction and RT-qPCR

Mononuclear cells were freshly isolated from bone marrow with ethylenediamine tetraacetic acid (EDTA) as coagulant from patients/healthy donors by standard density gradient centrifugation. Total RNA was extracted using TRIzol reagent (Invitrogen, Carlsbad, CA, USA) according to the manufacturer’s recommendations and reverse transcribed into cDNA using High Capacity cDNA Reverse Transcription Kit (Applied Biosystems, Foster City, CA, USA) [26]. TaqMan Gene Expression Assay was used for *SLC25A21* mRNA detection as previously described [26]. The standard curve was established from serial dilutions of plasmid expressing *ABL1* (Genechem). Then, the *SLC25A21* transcript level was calculated as the ratio of the *SLC25A21* copy number/*ABL1* copy number as previously described [27]. The primers and probes sequences of *SLC25A21* and *ABL1* are shown in Table S1. The *CXCL8* mRNA expression levels were quantified using SYBR Green Master Mix (YEASEN, Shanghai, China). Relative gene expression values were calculated using the 2^−ΔΔCt^ method normalizing to *GAPDH* expression. The primers sequences of *CXCL8* and *GAPDH* are listed in Table S1.

### Western blot analyses

Cells were harvested with RIPA lysis buffer (Beyotime) supplemented with phenylmethylsulfonylfluoride (PMSF, Biomed, Beijing, China) and phosphatase inhibitor cocktail (Biomed). The whole-cell protein lysates were quantified, subjected to SDS-polyacrylamide gel electrophoresis (SDS-PAGE) gels, and transferred to 0.45 μm polyvinylidene difluoride (PVDF) membrane (Millipore, Billerica, MA, USA). After blocking in 5% skim milk, the membrane was incubated with specific primary antibodies and corresponding secondary antibodies. The antibodies used included Cleaved-PARP, P53, Cleaved caspase3, p27 Kip1, p21 Waf1/Cip1, Cyclin D1, CDK4 (Cell Signaling Technology [CST], MA, USA, 1:1000), SLC25A21, cleaved caspase9 (Affinity Biosciences LTD, Jiangsu, China, 1:1000), α-Tublin (Proteintech, Wuhan, China, 1:100,000). BAX, BCL2 (Proteintech, Wuhan, China, 1:1000). HRP-labeled Goat Anti-Rabbit IgG(H + L), HRP-labeled Goat Anti-Mouse IgG(H + L) (Beyotime, Shanghai, China). The immunoreactive bands were detected using Super ECL Prime (US EVERBRIGHT, Suzhou, China) according to the manufacturer’s protocol.

### Cell proliferation

Cell proliferation was analyzed using the Cell Counting Kit-8 (CCK8, Dojin Laboratories, Kumamoto, Japan). A total of 1 × 10^4^ Kasumi-1 or THP-1 cells were seeded in 96-well plates with 100 μL medium containing 6 μg/mL Doxycycline. At the time points of 0, 24, 48, 72, and 96 h after seeding, the cells were respectively incubated with 10 μL CCK8 reagent for 3 hours, and the optical density (OD) was then detected spectrophotometrically at 450 nm. Experiments were performed in triplicate.

### Colony forming assay

The colony formation was assayed by sorting 6 × 10^3^ cells into individual 35 mm dish, each containing 1.1 mL of methylcellulose-based MethoCult medium (STEMCELLTM TECHNOLOGIES, Vancouver, British Columbia, Canada) and 6 μg/mL Doxycycline. A cell colony was defined as a cell formation of at least 30 cells. The surviving colonies were counted under an inverted microscope after 10 days of growing in a humid incubator. After 10 days of culture at 37 °C in a humidified 5% CO_2_ incubator, colonies were counted under the inverted microscope. All experiments were performed 3 times independently.

### Cell cycle and apoptosis analyses

Cells were seeded into six-well plates at a density of 1 × 10^5^ cells per mL. Then they were starved in serum-free medium for 24 h. After that, the medium was replaced with complete medium containing 6 μg/mL Doxycycline for an additional 72 hours. The Cell Cycle Staining Kit (Lianke Biotechnology, Hangzhou, China) was applied for cell cycle analyses. The Annexin V-APC/PI Apoptosis Kit (US EVERBRIGHT) was used to detect apoptotic cell death according to the manufacturer’s instructions. The cell cycle and apoptosis were examined by BD FACSCelesta^TM^ flow cytometry (*BD* Biosciences, California, USA).

### Xenograft tumor mouse model

Six-week-old male BALB/c nude mice (GemPharmatech, Nanjing, China) were used for the xenograft model. The nude mice were randomly assigned into two groups (CTRL, OE, *n* = 5 per group) and pre-treated with cyclophosphamide at 100 mg/kg/d via intraperitoneal injection for 2 days. Then the transfected cells were injected into the mice’s right flank. Doxycycline (1 mg/mL, Macklin Biochemical Co., Ltd, Shanghai, China) was supplemented in drinking water throughout the entire experiment. The tumor volume was measured every three days for 24 days using the following formula: volume (mm^3^) = (*L* × *I*^2^)/2, where *L* and *I* are the lengthiest and shortest diameters, respectively. At the end of the experiments, all mice were euthanized, and the tumors were collected, weighed, and photographed. The animal study was reviewed and approved by the Laboratory Animal Center of Zhengzhou University.

### Statistical analyses

*Pearson Chi-square test* or Fisher exact analysis, as appropriate, was applied for categorical data. Student’s *t*-test or Mann–Whitney *U*-test was used for continuous variables. Survival analysis was performed using the Kaplan–Meier method and estimated by Log-Rank test. In the ZZU cohort, patients were classified into the high-expression group and the low-expression group according to the cutoff value of *SLC25A21*. The association between *SLC25A21* transcript levels and OS was evaluated in *Cox* proportional hazard regression model. Variables with *P* < 0.2 in univariate analysis were included in the multivariable analysis model, and two-sided *P* < 0.05 (two-sided) was considered statistically significant (*P < 0.05; **P < 0.01; ***P < 0.001; ****P < 0.0001). The corresponding 95% confidence interval (CI) and hazard ratio (HR) were calculated. Statistical analysis was performed using Graphpad Prism™ 8.01 (San Diego, California, USA) and R (version 4.1.1, Auckland, NZ, USA, http://www.r-project.org/).

## Results

### Low expression of *SLC25A21* was a poor prognostic biomarker for AML patients

**Fig. 1:**
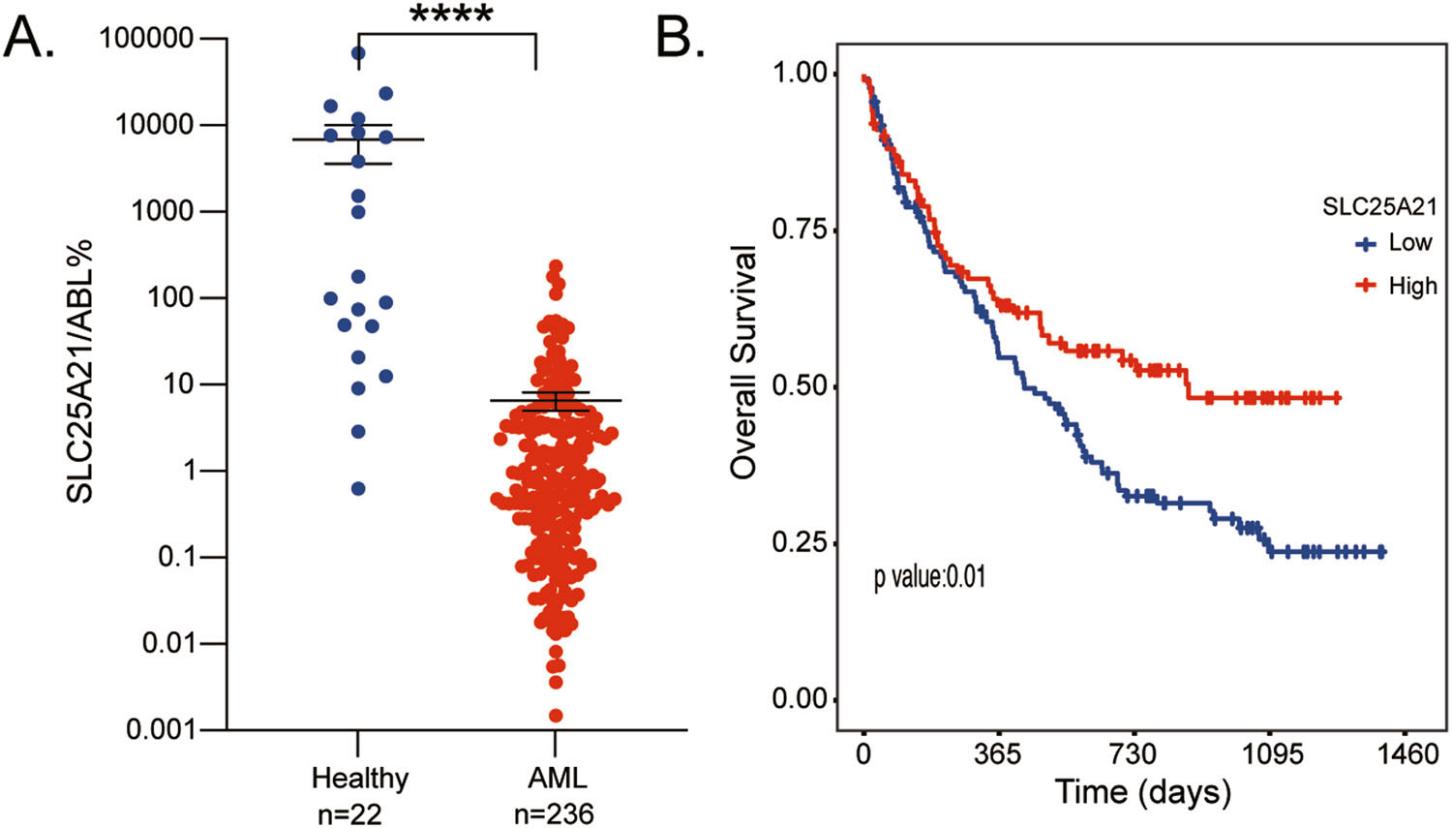
The downregulation of *SLC25A21* predicts poor prognosis in AML patients. **A** *SLC25A21* mRNA expression is significantly reduced in AML samples (*n* = 236) compared with normal bone marrow samples (*n* = 22). **B** Overall survival (OS) analysis of 236 AML subjects in our cohort. ****P < 0.0001.

To confirm whether *SLC25A21* was a potential biomarker for AML, we first examined the mRNA transcript levels of *SLC25A21* in the bone marrow of 236 newly diagnosed subjects with AML and 22 normal healthy individuals in our cohort. *SLC25A21* expression in AML patients was significantly lower than in healthy individuals (median 0.608% vs 138.500%, *P* < 0.001, Fig. 1A). Furthermore, we examined the role of *SLC25A21* in the prognosis of AML patients.

In this cohort, patients were classified into the low-*SLC25A21* expression group (<0.94%, *n* = 135) and high-*SLC25A21* expression group (≥0.94%, *n* = 101) according to the X-tile [28]. Subjects with high-*SLC25A21* expression showed a favorable 2-year OS rate compared to subjects with low *SLC25A21* expression (52.70%, 95% CI [43.24–64.23%] vs 32.63% [25.24–42.19%], *P* = 0.01; Fig. 1B). The baseline clinical characteristics of AML were described in detail in Table S2. Multivariate *Cox* regression showed that *SLC25A21* and transplant were independently associated with OS (Table 1).

**Table 1:** Univariate and multivariate analysis of overall survival in AML.

Characteristics	Univariate analysis			Multivariable analysis		
	HR	95% CI	*P* value	HR	95% CI	*P* value
**High*-SLC25A21*** **group**	**0.63**	**0.44**–**0.9**	**0.011**	**0.59**	**0.38**–**0.89**	**0.013**
Age (≥60 years)	2.27	1.54–3.34	<0.0001	1.58	0.99–2.51	0.054
**Transplant**	**0.22**	**0.11**–**0.45**	**<0.0001**	**0.21**	**0.1**–**0.47**	**0.0001**
Risk						
Intermediate vs. Favorable	2.01	1.18–3.42	0.010	1.70	0.93–3.10	0.086
Poor vs. Favorable	2.77	1.63–4.70	0.0001	1.93	0.99–3.77	0.054
Sex (male vs. female)	0.85	0.60–1.19	0.333	NA	NA	NA
HGB (≥80 g/L)	1.03	0.74–1.45	0.844	NA	NA	NA
LDH (≥500 U/L)	1.03	0.73–1.44	0.885	NA	NA	NA
BM-blast (≥65%)	1.04	0.74–1.46	0.807	NA	NA	NA
WBC (≥20 × 10^9^/L)	1.30	0.92–1.82	0.135	0.94	0.64–1.38	0.753
PLT (≥45 × 10^9^/L)	1.08	0.77–1.51	0.670	NA	NA	NA
PB (≥50%)	0.97	0.68–1.36	0.844	NA	NA	NA
*SRSF2*	2.73	1.42–5.27	0.003	1.95	0.86–4.43	0.110
*RUNX1*	2.19	1.15–4.19	0.018	1.35	0.65–2.81	0.427
*FLT3*	1.53	1.05–2.23	0.026	0.53	0.07–3.99	0.539
*ETO*	0.50	0.27–.93	0.029	0.94	0.43–3.99	0.880
*KIT*	0.43	0.19–0.97	0.041	0.58	0.22–1.56	0.282
*FLT3_ITD*	1.49	1.01–2.19	0.043	2.1	0.28–15.95	0.473
*CBL*	2.15	0.79–5.83	0.133	1.18	0.34–4.11	0.797
*FLT3_TKD*	2.14	0.79–5.81	0.135	3.52	0.61–20.36	0.159
*U2AF1*	1.63	0.86–3.12	0.136	1.72	0.80–3.73	0.168
*NRAS*	0.75	0.49–1.14	0.177	0.91	0.58–1.44	0.697
*DNMT3A*	1.34	0.84–2.14	0.216	NA	NA	NA
*NPM1*	1.31	0.84–2.03	0.233	NA	NA	NA
*CEBPA*	0.78	0.51-1.18	0.239	NA	NA	NA
*IDH2*	1.35	0.72–2.50	0.347	NA	NA	NA
*IDH1*	0.74	0.37–1.45	0.373	NA	NA	NA
*ASXL1*	0.83	0.54–1.27	0.393	NA	NA	NA
*SETBP1*	1.73	0.43–7.00	0.442	NA	NA	NA
*MLL*	0.61	0.15–2.45	0.481	NA	NA	NA
*JAK2*	1.59	0.39–6.43	0.517	NA	NA	NA
*TP53*	0.72	0.18–2.93	0.649	NA	NA	NA
*PHF6*	1.55	0.22–11.15	0.664	NA	NA	NA
*WT1*	0.93	0.66–1.31	0.677	NA	NA	NA
*ETV6*	0.81	0.20–3.27	0.766	NA	NA	NA
*TET2*	0.97	0.69–1.36	0.868	NA	NA	NA
*EZH2*	0.89	0.12–6.41	0.911	NA	NA	NA
*CBFβ*	0	0-Inf	0.994	NA	NA	NA

Bold value indicates *P* value that remained significant in multivariate analysis.

*WBC* white blood cell counts, *HGB* hemoglobin, *PLT* platelet, *LDH* lactate dehydrogenase, *BM* bone marrow, *PB* peripheral blood.

### Overexpression of *SLC25A21* inhibited cell proliferation and colony formation of AML cells

**Fig. 2:**
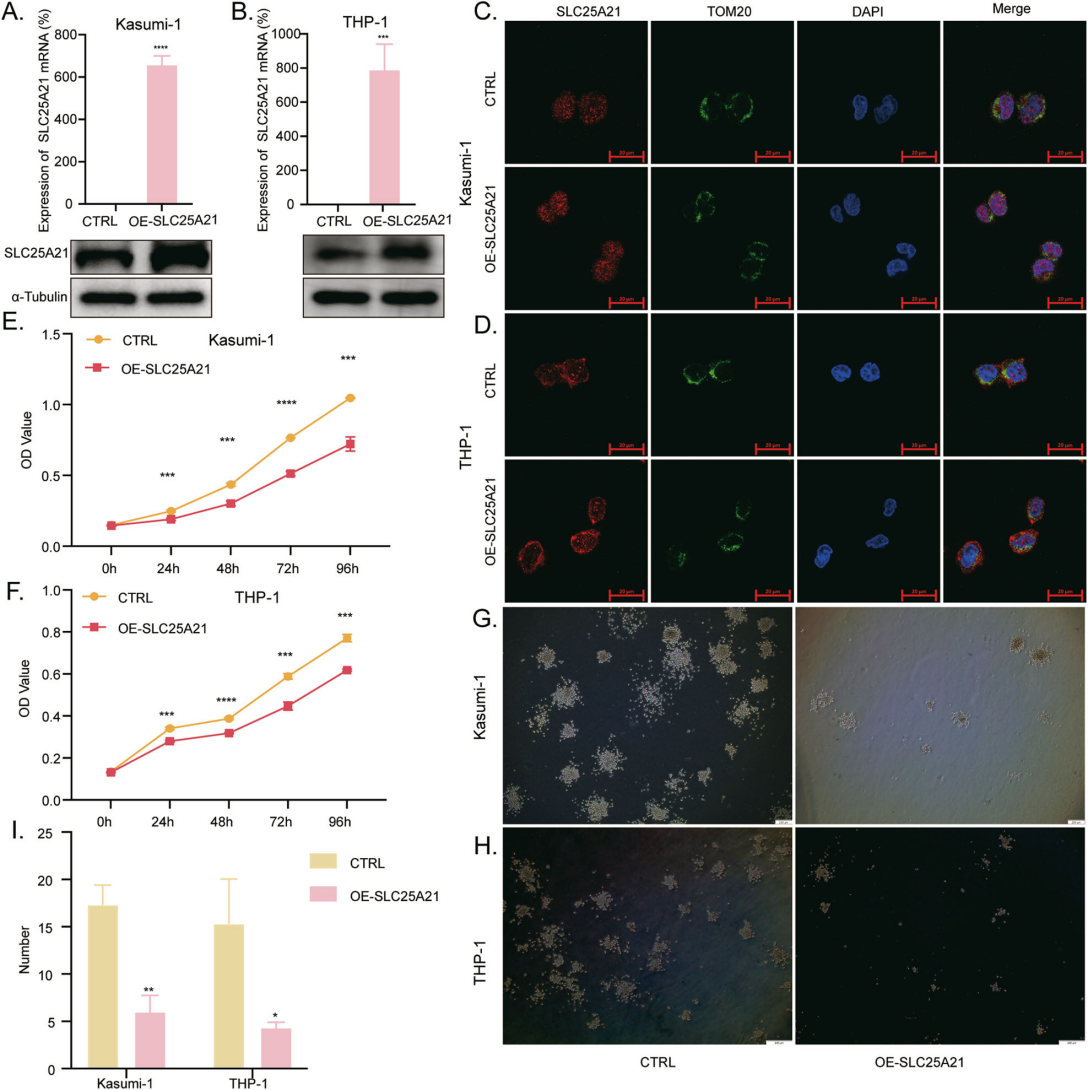
*SLC25A21* overexpression inhibited cell proliferation and colony formation in AML cells. **A**, **B** RT-qPCR and Western blot analysis of the overexpression efficiency of SLC25A21 in Kasumi-1 and THP-1 cell lines. **C**, **D** Immunofluorescence provides information about SLC25A21 expression and subcellular localization in Kasumi-1 and THP-1 cell lines. **E**, **F** Kasumi-1 and THP-1 cell lines were analyzed for cell proliferation by using CCK8 assay. **G**–**I** The effect of *SLC25A21* overexpression on colony-forming ability was detected by colony-formation assays. CTRL, control; OE, overexpression; *P < 0.05 compared with CTRL cells; **P < 0.01 compared with CTRL cells; ***P < 0.001 compared with CTRL cells; ****P < 0.0001 compared with CTRL cells; Error bars indicate the standard deviation.

We next set out to identify the potential biological roles of *SLC25A21* in vitro. We used lentivirus-mediated infection to stably overexpress *SLC25A21* in AML cell lines Kasumi-1 and THP-1. The overexpression efficiency was determined by RT-qPCR and Western Blot (WB). Compared with the control group, *SLC25A21* mRNA increased by more than 600-fold in both Kasumi-1 (Fig. 2A) and THP-1 (Fig. 2B) cell lines, and there was also a significant increase in SLC25A21 protein levels (Fig. 2A, B). Immunofluorescent assays showed that SLC25A21 protein not only co-colocalized with TOM20, a mitochondrial marker, but also to the nucleus (Fig. 2C, D). CCK8 assays indicated that the cell proliferation was significantly decreased with *SLC25A21* overexpression in both Kasumi-1 (Fig. 2E) and THP-1 (Fig. 2F) cell lines. Consistently, colony formation with *SLC25A21* overexpression also significantly reduced in both cell types (Fig. 2G–I).

### Overexpression of *SLC25A21* Inhibit Cell Cycle of AML Cells

**Fig. 3:**
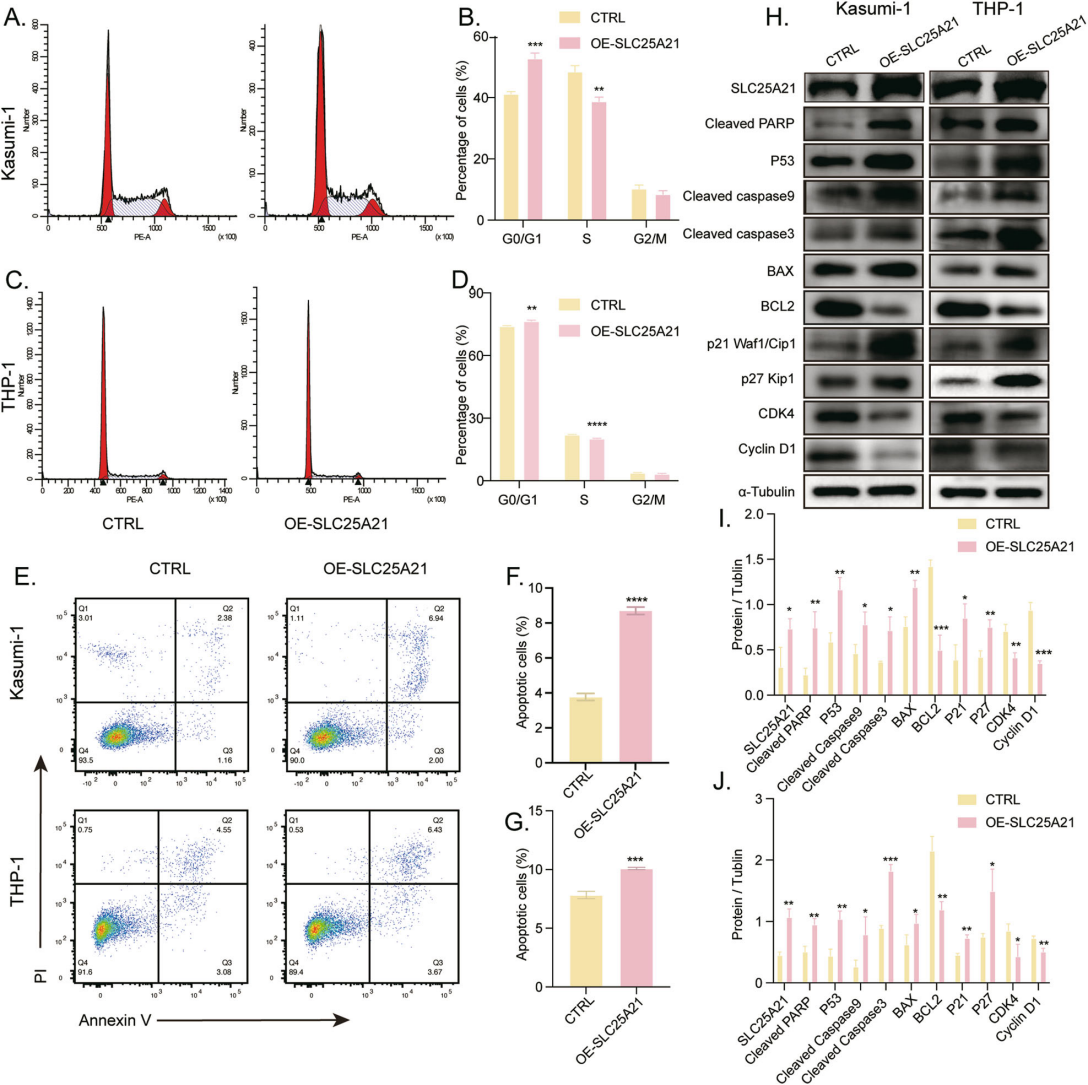
Effect of SLC25A21 on the cell cycle and apoptosis of AML cells in vitro. **A**–**D** The cell cycle of SLC25A21 overexpression cells compared with control was analyzed by Propidium iodide (PI) staining‐based flow cytometry. **E**–**G** The percentage of apoptotic cells was determined by flow cytometric analysis at 72 h. **H** The expression levels of cell cycle-related proteins and apoptosis-related proteins were analyzed by Western blotting. The protein gray values in (**I**) Kasumi-1 and (**J**) THP-1 cell lines were quantitated by Image J (*n* = 3 samples per group). CTRL, control; OE, overexpression; *P < 0.05 compared with CTRL cells; **P < 0.01 compared with CTRL cells; ***P < 0.001 compared with CTRL cells; ****P < 0.0001 compared with CTRL cells; Error bars indicate the standard deviation.

To check whether reduction in cell proliferation and colony formation is associated with cell cycle inhibition, we evaluated the role of *SLC25A21* in the cell cycle progression using flow cytometric cell cycle analysis. *SLC25A21* overexpression increased the number of cells in the G0/G1 phase, which reflected G0/G1 arrest. Consistently, the cell population in the S phase decreased, indicating impaired cell cycle progression (Fig. 3A–D). We then compared the specific protein changes associated with cell cycle. Results showed that in *SLC25A21*-overexpressed cells, the expression levels of G1-phase regulators proteins, CDK4 and Cyclin D1, were downregulated, while the CDK inhibitors p21 Waf1/Cip1and p27 Kip1 were correspondingly upregulated (Fig. 3H–J).

### *SLC25A21* overexpression induced apoptosis via mitochondrial apoptotic signaling pathway in AML cells

Since cell cycle inhibition could lead to cell death, we examined the relation between *SLC25A21* overexpression and cell apoptosis. Flow cytometry using Annexin V/PI staining showed more apoptotic cells in *SLC25A21* overexpressed groups (Fig. 3E–G). It has been identified that *SLC25A21* induced caspase-dependent apoptosis and the loss of mitochondrial membrane potential [13]. Therefore, we compared the protein level changes in AML cells overexpressing SLC25A21 that are associated with the mitochondrial apoptotic pathway. Cleaved PARP, P53, Cleaved caspase 9, Cleaved caspase 3 and BAX were significantly increased, and BCL2 expression was decreased with the overexpression of SLC25A21 (Fig. 3H–J). Moreover, an increase in cellular ROS was observed in Kasumi-1 and THP-1 cells with the overexpression of *SLC25A21* (Fig. S1).

### Overexpression of *SLC25A21* inhibited tumor growth in vivo

**Fig. 4:**
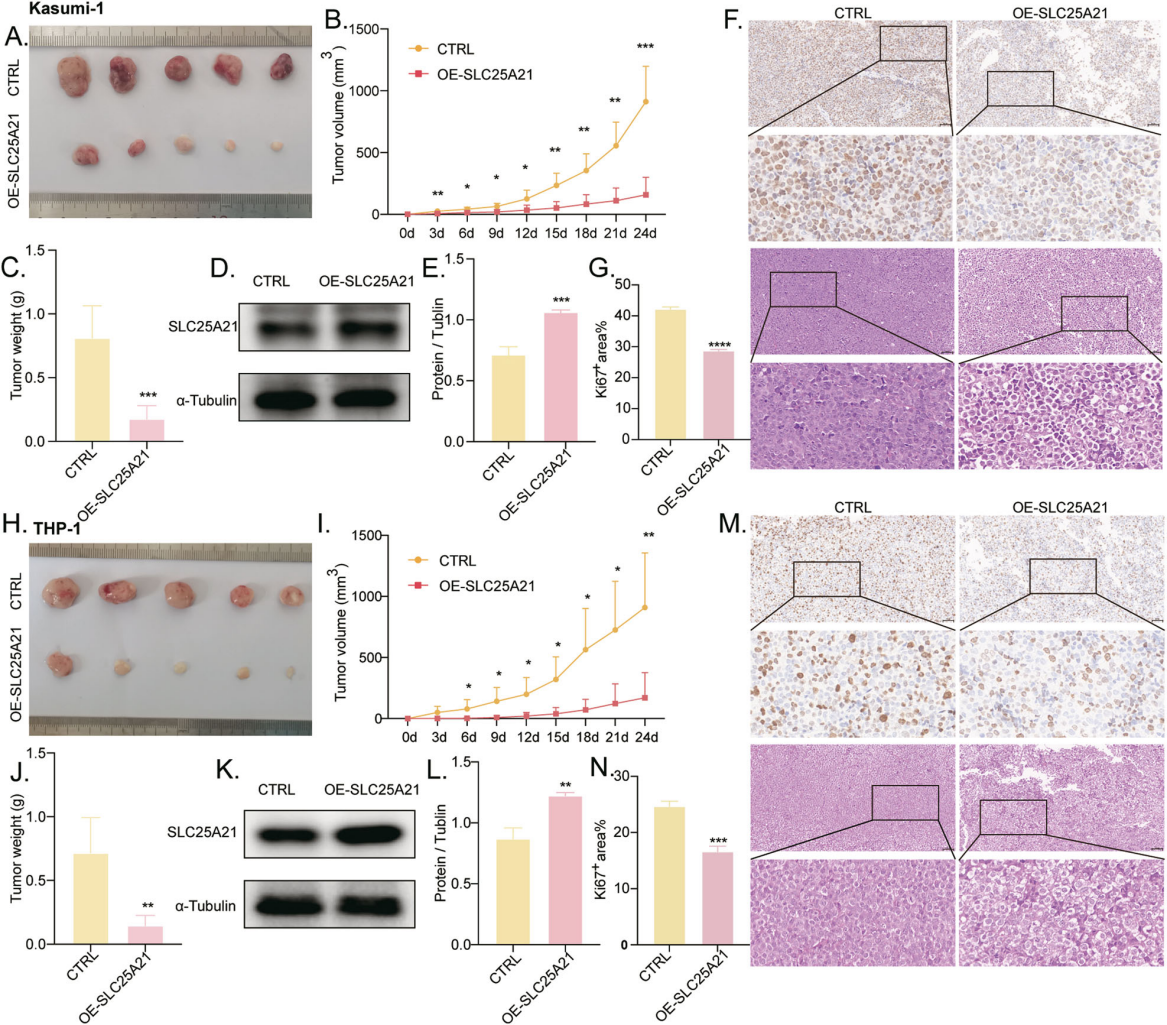
SLC25A21 overexpression repressed the progression of AML (**A**–**G**: Kasumi-1; **H**–**N**: THP-1) in vivo. (**A**–**C**, **H**–**J**) SLC25A21 overexpression inhibited tumor growth in the xenograft nude mouse model (*n*= 5 per group). **D**, **E**, **K**, **L** The expression of SLC25A21 in tumor tissues by Western blot. **F**, **M** HE staining and Ki67 expression of xenograft tumors. **G**, **N** Statistical analysis of Ki67-positive area (*n* = 3). CTRL, control; OE overexpression; *P < 0.05 compared with CTRL cells; **P < 0.01 compared with CTRL cells; ***P < 0.001 compared with CTRL cells; ****P < 0.0001 compared with CTRL cells; Error bars indicate the standard deviation.

The in vitro evidence indicated that *SLC25A21* overexpression inhibit AML cell proliferation. Thus, we tested whether the overexpression of *SLC25A21* in vivo suppressed tumor growth inhibition, using xenograft experiments (Fig. 4A–C, H–J). We found that the tumor volume and mass in nude mice subcutaneously injected with *SLC25A21*-overexpressing Kasumi-1 (Fig. 4A–C) and THP-1 (Fig. 4H–J) cells were significantly reduced compared with the control group. Additionally, Ki67 immunohistochemical staining in xenograft tumors showed that *SLC25A21*-overexpressing tissues had significantly less Ki67 staining than the control group (Fig. 4F, G, M, N). At the same time, HE staining of the subcutaneous tumor tissues (Fig. 4F, M) also confirmed that the overexpression of   *SLC25A21* was associated with inhibited cellular proliferation. The protein levels of *SLC25A21* from subcutaneous tumor tissues of nude mice was also examined using WB (Fig. 4D, E, K, L). Collectively, these results suggested that *SLC25A21* is able to suppress tumor growth in vivo.

### *SLC25A21* overexpression inhibited AML progression by downregulating *CXCL8* expression

**Fig. 5:**
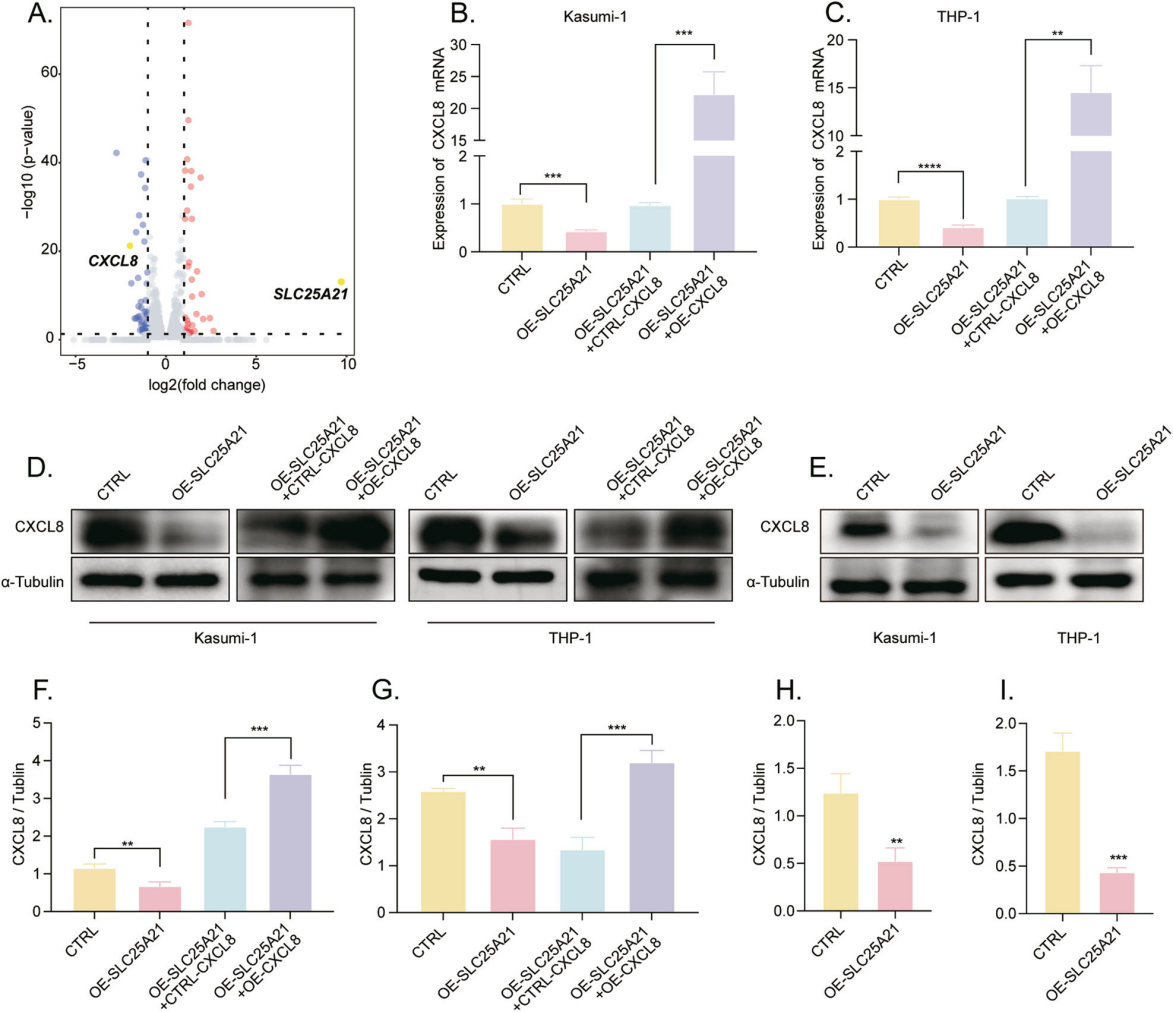
*SLC25A21* inhibited the expression of *CXCL8.* **A** Volcano plot of differential gene expression in *SLC25A21* stably transfected AML cell line Kasumi-1 (*n* = 3 per group). Expression levels of CXCL8 following SLC25A21 overexpression in (**B**, **D**) Kasumi-1 and (**C**, **D**) THP-1 cell lines. **B**–**D** RT-qPCR and Western blot analysis of the overexpression efficiency of CXCL8 in SLC25A21 stably transfected AML cell lines. The protein gray values were quantitated by Image J (*n* = 3 samples per group) in (**F**) Kasumi-1 and (**G**) THP-1 cell lines. **E** The expression of CXCL8 in tumor tissues by western blot. The protein gray values were quantitated by Image J (*n* = 3 samples per group) in (**H**) Kasumi-1 and (**I**) THP-1 group. CTRL control; OE overexpression; **P < 0.01 compared with CTRL cells; ***P < 0.001 compared with CTRL cells; ****P < 0.0001 compared with CTRL cells; Error bars indicate the standard deviation.

**Fig. 6:**
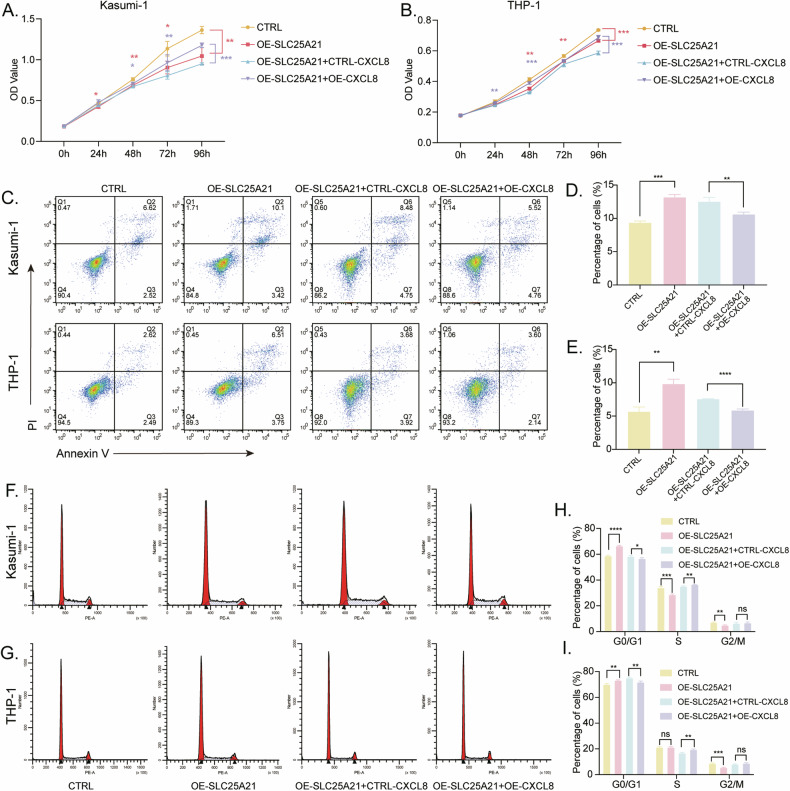
The suppressive effect of *SLC25A21* on AML progression was rescued by *CXCL8.* **A**, **B** Cell proliferation was detected by CCK8 assays. The distribution of AML cell lines in the apoptosis (**C**–**E**) and each phase of cell cycle (**F**–**I**) were calculated in each group. CTRL control, OE overexpression, ns nonsignificant; *P < 0.05 compared with CTRL cells; **P < 0.01 compared with CTRL cells; ***P < 0.001 compared with CTRL cells; ****P < 0.0001 compared with CTRL cells; Error bars indicate the standard deviation.

To understand mechanistically how *SLC25A21* affects disease progression in AML, total cell extracts from the control and *SLC25A21* overexpression cells were prepared and subjected to RNA-seq. The volcano plot showed that a total of 36 genes were upregulated (*P*-adjust < 0.05, Log2fold change > 1), and 48 genes were downregulated, including *CXCL8* (*P*-adjust < 0.05, Log2fold change < −1) (Fig. 5A). We found that *CXCL8* expression was inhibited by *SLC25A21* overexpression in vitro and in vivo (Fig. 5D–I). Since *CXCL8* plays an important role in hematologic malignancies [19–22], we focused on *CXCL8* for further study. When *CXCL8* is overexpressed in *SLC25A21* stably-overexpressed AML cells, we first confirmed the overexpression of *CXCL8* at the level of mRNA and protein (Fig. 5B–D, F, G). CCK-8 assays showed that the overexpression of *SLC25A21* inhibited the proliferation of Kasumi-1 and THP-1 cells, but the overexpression of *CXCL8* reversed the *SLC25A21*-induced proliferation inhibition of AML cells (Fig. 6A, B). Overexpression of *SLC25A21* caused a significant increase in cellular apoptosis that was substantially rescued by overexpressing *CXCL8* (Fig. 6C–E). Similarly, the overexpression of *CXCL8* could partially reverse the inhibition effects of *SLC25A21* overexpression on the cell cycle progression in AML cell lines (Fig. 6F-I). Together, these findings confirmed that *SLC25A21* regulated the biological function of AML cells through downregulating *CXCL8*.

## Discussion

In this study, we found that *SLC25A21* expression was significantly suppressed in adult AML patients compared with healthy donors. The low expression level of *SLC25A21* was significantly associated with a poor prognosis in AML patients. Furthermore, we demonstrated that the overexpression of *SLC25A21* could inhibit AML cell proliferation and colony formation, induce apoptosis and arrest cell cycle via targeting downregulation of *CXCL8* expression. The in vivo data further supported the in vitro results. These findings indicate that *SLC25A21* is a novel biomarker for AML and has great potentials for targeted treatment.

In recent years, the roles of *SLC25A21* in some cancers have been reported. For example, a recent study by Yong Wang et al. demonstrated that *SLC25A21* was downregulated in bladder cancer [13]. The low expression of *SLC25A21* promotes BCa progression in ROS-mediated mitochondrion-dependent apoptosis pathway. They further identified that *SLC25A21* dysregulation plays an important role in rewiring tumor metabolism in KRAS-mutant CRC [14]. DNA promoter hypermethylation is critical in the downregulation of *SLC25A21* expression. *SLC25A21* hypermethylation was correlated with poorer prognosis of Glioma patients [12]. Wenjun Wang et al. provided a comprehensive pan-cancer analysis of *SLC25A21* expression based on the TCGA data [29]. They found that *SLC25A21* was downregulated in a variety of malignancies, especially in AML. This finding was also corroborated by the analysis of the GSE13159 and GSE12417 data sets. Our clinical data also suggested that low *SLC25A21* expression was a potential indicator for a poor prognosis in AML patients. However, the biological functions and mechanisms of *SLC25A21* in AML remain largely unknown. Our findings shed light on the mechanisms of *SLC25A21* regulation in AML. In our study, we have identified the biological functions of *SLC25A21* in cell proliferation, apoptosis, and cell cycle regulation. These findings were consistent with the analysis of *SLC25A21* expression in AML patients, suggesting that the overexpression of *SLC25A21* in AML may serve as a potential tumor-suppressing factor by inhibiting cell proliferation, facilitating cell apoptosis, and inducing cell cycle arrest.

Mitochondria are integral players in the production of ROS and regulation of apoptosis [30]. When cells experience DNA damage or other stress, P53 is activated and translocates to the surface of mitochondria. Then P53 directly activates the proapoptotic BCL-2 family proteins Bax [31]. When BAX activates the intrinsic apoptotic pathway, cytochrome c (Cyto c) and other proapoptotic mediators are released from the intermembrane space (IMS) [32]. Once released to the cytosol, Cyto c could bind to Apaf-1, dATP and pro-caspase-9 to form an oligomeric complex [33]. The apoptosome then activates caspase-9 and its downstream caspases-3, which ultimately leads to apoptosis [34]. We found that overexpression of *SLC25A21* increased ROS production (Fig. S1). We also observed an increased expression of Cleaved PARP, P53, Cleaved caspase 9, Cleaved caspase 3 and BAX, as well as a reduced expression of BCL2 with the overexpression of *SLC25A21*. These results proved that *SLC25A21* could inhibit the proliferation and induce apoptosis via activating the mitochondrial pathway in AML cells. Furthermore, the overexpression of *SLC25A21* caused G0/G1 cell cycle arrest via the p27 Kip1/Cyclin D1-CDK4 pathway. The p27 Kip1 is a cyclin-dependent kinase inhibitor, which induces cell cycle arrest in the G0/G1 phase [35–38]. It also inhibits CDK4/cyclin D1 activity [37].

To investigate how *SLC25A21* contributes to AML development, we performed RNA-seq in stable *SLC25A21* overexpression cells. The RNA-seq analysis showed a decreased expression of *CXCL8* (also known as *IL8*). It has been reported that the expression of *CXCL8* was promoted by monocarboxylate transporter 1 (MCT1)-mediated lactate uptake in endothelial cells [16]. Lactate-dependent induction of *CXCL8* expression promoted tumor growth in vivo. *SLC25A21* could impact glutamine (Gln)-derived α-KG efflux, Gln metabolism, and GTP production. Also, α-KG could suppress the expression of *CXCL8*.^16^*CXCL8* is implicated in various cellular processes, including tumor progression. It has been suggested as a biomarker for non-small cell lung cancer [39]. Some studies have indicated that *CXCL8* is associated with poor prognosis in colorectal cancer [40, 41]. A study demonstrated that the Hsp60-IL-8 axis could represent a therapeutic target in colorectal and prostate malignancies [42]. Xiao et al. found that *CXCL8* inhibits anoikis of CRC cells by regulating the mitochondrial apoptotic pathway [40]. Schinke et al. found that the IL8-CXCR2 pathway is dysregulated in AML and MDS stem cells, which could be a novel therapeutic target [19]. The inhibition of *CXCL8*/*CXCR2* presents a promising therapeutic opportunity for intercepting the progression of myelofibrosis in myeloproliferative neoplasms [43]. Hu et al. found that *CXCL8* was correlated with AML relapse, and the knockdown of *CXCL8* reduced the proliferation of AML cell lines [14]. Thus, We further showed proof by performing rescue experiments. *CXCL8* was overexpressed in AML cells with *SLC25A21* stable overexpression. Subsequently, cell proliferation, cell cycle, and apoptosis were measured. The primary biological functions of *SLC25A21* were suppressed after *CXCL8* overexpression in vitro, confirming the tumor-promoting action of *CXCL8*. The exact mechanism by which the *SLC25A21* regulates *CXCL8* in AML remains unclear, and much work needs to be done in our subsequent studies.

## Conclusions

In summary, our study reveals that low *SLC25A21* expression is associated with poor prognosis in AML patients. More importantly, the overexpression of *SLC25A21* represses AML cell proliferation and cell cycle progression, and promotes apoptosis through *CXCL8* downregulation. Our data suggest that *SLC25A21* may be a potential diagnostic marker for prognosis and a personalized therapeutic target for AML in the future.

## Supplementary information

Supplementary materials and methods

Original WB data

check list

## Data Availability

The datasets generated and analyzed during the current study are not publicly available due to patient privacy considerations, but are available from the corresponding author on reasonable request (Shujuan Wang: fccwangsj1@zzu.edu.cn).
