# Supplementary material for: SLC25A21 correlates with the prognosis of adult acute myeloid leukemia through inhibiting the growth of leukemia cells via downregulating CXCL8
Source: Cell Death Dis. 2024 Dec 20;15(12):921. doi: 10.1038/s41419-024-07308-y (PMC11662024; doi:10.1038/s41419-024-07308-y)
Supplement: Supplementary file 1 — Supplementary materials and methods [file 41419_2024_7308_MOESM1_ESM.docx]

**Supplementary materials and methods****Cell Lines and Reagents**

Human AML cell line Kasumi-1 was originally obtained from American Type Culture Collection (ATCC; Manassas, VA, USA). Human AML cell line THP-1 was purchased from the Shanghai Cell Bank of the Chinese Academy of Sciences (Shanghai, China). Prior to the experiment, we performed STR profiling on the two cell lines to ensure their authenticity and confirmed that there was no mycoplasma contamination. The cell lines were cultured in 90% Roswell Park Memorial Institute (RPMI**)** 1640 media containing 10% Fetal bovine serum (FBS) (Gibco, Billings, MT, USA), and 1% penicillin-streptomycin (Gibco, Billings, MT, USA). All cells were maintained in a humidified atmosphere at 37°C with 5% CO_2_ and air.

**Next-Generation Sequencing**

The application of Next-Generation sequencing was utilized to evaluate the mutational hotspots of genes. The Rightongene AML/MDS/MPN Sequencing Panel (Rightongene, Shanghai, China) was performed on Illumina MiSeq System (Illumina, San Diego, CA) high-throughput sequencing platform. Details of the variant calling, filtering, and annotation have been published.^1^

**Cytogenetics and fusion genes analysis.**

According to the International System for Human Cytogenetic Nomenclature, the chromosomal banding analyses were conducted using G-banding techniques. The detection of fusion genes was conducted using Multiplex real-time quantitative polymerase chain reaction (RT-qPCR) Fusion Gene Kits (Rightongene) through the application of RT-qPCR.

**Immunofluorescence confocal microscopy**

Cells in T25 flasks were fixed in 4% paraformaldehyde (PFA) for 20 min at room temperature (RT). After washing three times by Immunol staining wash buffer (Beyotime, Shanghai, China), cells were blocked in the blocking buffer (QuickBlock™ Blocking buffer for immunol staining, Beyotime, Shanghai, China) for 15 minutes. Followed by washing, cells were incubated overnight at 4°C with primary SLC25A21 antibody (Affinity Biosciences LTD, Jiangsu, China) and TOM20 antibody (Proteintech, Wuhan, China). Then, the cells were incubated with secondary antibody Cy3 conjugated Goat Anti-Rabbit IgG (H+L) (Servicebio, Wuhan, China) and Fluorescein (FITC) –conjugated Affinipure Goat Anti-Mouse IgG (H+L) for 2h at RT. Subsequently, the cells were counterstained with DAPI (Solaibao Biotechnology, Beijing, China), and imaged under confocal laser scanning microscopy (Zeiss, Oberkochen, Germany).

**H&E staining and Immunohistochemical Assays**

Hematoxylin and eosin staining (H&E staining) and Immunohistochemistry (IHC) were performed as we have previously described^2^. The following antibodies were used: Ki67 (Servicebio, Wuhan, China), and HRP conjugated Goat Anti-Rabbit IgG (H+L) (Servicebio, Wuhan, China).

**Measurement of intracellular reactive oxygen species (ROS)**

Briefly, cells were resuspended in serum-free medium, and labeled with 5 μmol/L H2DCFDA (GLPBIO, Montclair, CA, USA). The cell suspension was incubated at 37℃ for 20 minutes and blended every 4 min in the dark. Photomicrographs were taken using confocal laser scanning microscopy (Zeiss, Oberkochen, Germany). Fluorescence intensity served as a measure of ROS levels within the cell.

**Supplementary References**

1. Yu J, Li Y, Li T, Li Y, Xing H, Sun H*, et al.* Gene mutational analysis by NGS and its clinical significance in patients with myelodysplastic syndrome and acute myeloid leukemia. *Exp Hematol Oncol* 2020, **9:** 2.

2. Liu Y, Chen Y, Liu Y, Li M, Zhang Y, Shi L*, et al.* Downregulation of SMIM3 inhibits growth of leukemia via PI3K-AKT signaling pathway and correlates with prognosis of adult acute myeloid leukemia with normal karyotype. *J Transl Med* 2022, **20**(1)**:** 612.

**
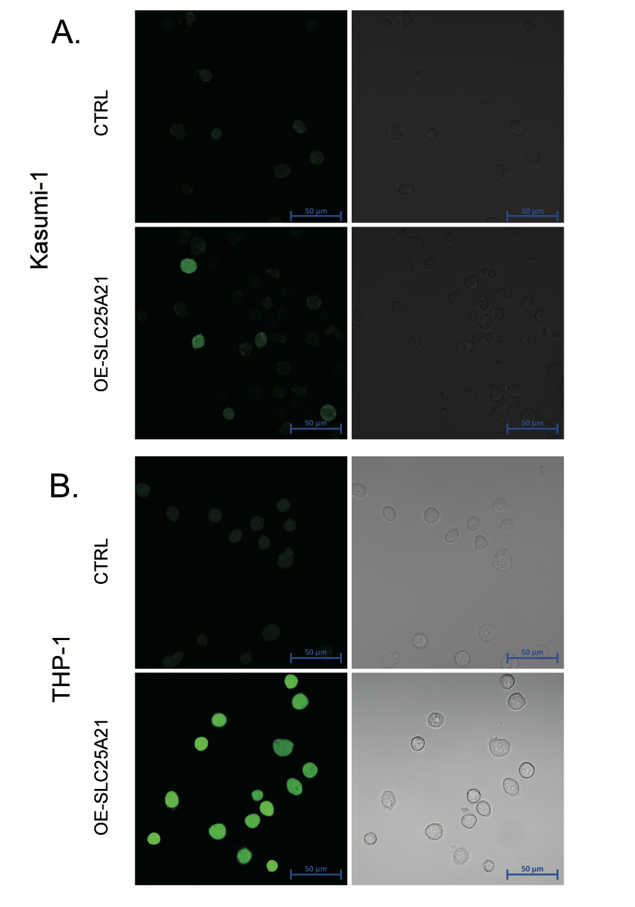
**

**Figure S1.**

Results of ROS production in (A) Kasumi-1 and (B) THP-1 cell lines.

**Table** **S1.** **Sequences of primers and probes used in this study**

| Name | Sequence (5'-3') |
| --- | --- |
| *SLC25A21*-Forward primer | GAAGTCAGCTTAGTGCGCGA |
| *SLC25A21*-Reverse primer | ATCTAGGGGGTGCATCAGGC |
| *SLC25A21*-Probe | 6-FAM-TGGCCGGTGGTTCTGCAGGTCTTGT-BHQ1 |
| *ABL1*-Forward primer | TGGAGATAACACTCTAAGCATAACTAAAGGT |
| *ABL1*-Reverse primer | GATGTAGTTGCTTGGGACCCA |
| *ABL1*-Probe | FAM-CCATTTTTGGTTTGGGCTTCACACCATT-TAMARA |
| *CXCL8*-Forward primer | CATACTCCAAACCTTTCCACCC |
| *CXCL8*-Reverse primer | CAAAAACTTCTCCACAACCCTCTG |
| *GAPDH*-Forward primer | GGAAGCTTGTCATCAATGGAAATC |
| *GAPDH*-Reverse primer | TGATGACCCTTTTGGCTCCC |

**Table S2. Relationship between the Transcription Levels of SLC25A21 and Clinical Characteristics in AML.**

| Characteristic | Total | L-SLC25A21 | H-SLC25A21 | *P*-value |
| --- | --- | --- | --- | --- |
|  | N=236 | N=135 | N=101 |  |
| Age (years), median (quartile) | 46 (32-55) | 46 (32-53) | 46 (33-56) | 0.781 |
| Female | 119 (50.42%) | 70 (51.85%) | 49 (48.51%) | 0.707 |
| Risk group n(%) |  |  |  | 0.383 |
| Poor | 92 (38.98%) | 50 (37.04%) | 42 (41.58%) |  |
| Intermediate | 96 (40.68%) | 60 (44.44%) | 36 (35.64%) |  |
| Favorable | 48 (20.34%) | 25 (18.52%) | 23 (22.77%) |  |
| FAB, n (%) |  |  |  | 0.592 |
| M0 | 15 (6.36%) | 10 (7.41%) | 5 (4.95%) |  |
| M1 | 11 (4.66%) | 7 (5.19%) | 4 (3.96%) |  |
| M2 | 112 (47.46%) | 60 (44.44%) | 52 (51.49%) |  |
| M4 | 30 (12.71%) | 16 (11.85%) | 14 (13.86%) |  |
| M5 | 58 (24.58%) | 34 (25.19%) | 24 (23.76%) |  |
| M7 | 8 (3.39%) | 7 (5.19%) | 1 (0.99%) |  |
| Unclassified | 2 (0.85%) | 1 (0.74%) | 1 (0.99%) |  |
| WBC, ×10^9^/L, median (quartile) | 24.83 (6.88-66.47) | 40.90 (12.18-97.78) | 11.11 (3.80-40.90) | **<0.001** |
| HGB, g/L, median (quartile) | 79.00 (65.00-93.25) | 75.10 (62.50-92.00) | 81.00 (68.00-95.00) | **0.037** |
| PLT, ×10^9^/L, median (quartile) | 44.00 (19.00-77.00) | 34.00 (15.00-57.50) | 56.00 (29.00-87.00) | **<0.001** |
| LDH U/L, median (quartile) | 459.00 (274.00-951.75) | 513.00 (301.50-1066.50) | 353.00 (246.00-740.00) | **0.002** |
| PB(%),median (quartile) | 62.50 (27.75-86.00) | 74.00 (41.50-88.00) | 48.00 (17.00-80.00) | **<0.001** |
| BM-blast(%), median (quartile) | 64.20 (42.80-83.53) | 70.00 (49.90-84.00) | 57.60 (36.40-78.40) | **0.007** |
| Karyotype |  |  |  | 0.966 |
| abnormal | 141 (59.75%) | 80 (59.26%) | 61 (60.40%) |  |
| normal | 95 (40.25%) | 55 (40.74%) | 40 (39.60%) |  |
| Mutations, n(%) |  |  |  |  |
| *FLT3* | 55 (23.31%) | 43 (31.85%) | 12 (11.88%) | **0.001** |
| *FLT3_ITD* | 50 (21.19%) | 43 (31.85%) | 7 (6.93%) | **<0.001** |
| *FLT3_TKD* | 6 (2.54%) | 1 (0.74%) | 5 (4.95%) | 0.087 |
| *JAK2* | 3 (1.27%) | 0 (0.00%) | 3 (2.97%) | 0.077 |
| *U2AF1* | 15 (6.36%) | 5 (3.70%) | 10 (9.90%) | 0.097 |
| *TP53* | 6 (2.54%) | 1 (0.74%) | 5 (4.95%) | 0.087 |
| *WT1* | 148 (62.71%) | 87 (64.44%) | 61 (60.40%) | 0.617 |
| *MLL* | 6 (2.64%) | 1 (0.78%) | 5 (5.10%) | 0.087 |
| *ETO* | 28 (12.33%) | 17 (13.08%) | 11 (11.34%) | 0.850 |
| *CBFβ* | 12 (5.29%) | 7 (5.38%) | 5 (5.15%) | 1.000 |
| *TET2* | 114 (48.31%) | 66 (48.89%) | 48 (47.52%) | 0.940 |
| *CEBPA* | 50 (21.19%) | 30 (22.22%) | 20 (19.80%) | 0.772 |
| *ASXL1* | 50 (21.19%) | 23 (17.04%) | 27 (26.73%) | 0.100 |
| *NRAS* | 52 (22.03%) | 32 (23.70%) | 20 (19.80%) | 0.578 |
| *KIT* | 19 (8.05%) | 11 (8.15%) | 8 (7.92%) | 1.000 |
| *NPM1* | 36 (15.25%) | 22 (16.30%) | 14 (13.86%) | 0.740 |
| *DNMT3A* | 33 (13.98%) | 20 (14.81%) | 13 (12.87%) | 0.813 |
| *IDH2* | 18 (7.63%) | 8 (5.93%) | 10 (9.90%) | 0.373 |
| *IDH1* | 19 (8.05%) | 7 (5.19%) | 12 (11.88%) | 0.103 |
| *RUNX1* | 12 (5.08%) | 6 (4.44%) | 6 (5.94%) | 0.827 |
| *SRSF2* | 13 (5.51%) | 3 (2.22%) | 10 (9.90%) | **0.023** |
| *ETV6* | 4 (1.69%) | 2 (1.48%) | 2 (1.98%) | 1.000 |
| *EZH2* | 2 (0.85%) | 0 (0.00%) | 2 (1.98%) | 0.182 |
| *SETBP1* | 2 (0.85%) | 2 (1.48%) | 0 (0.00%) | 0.508 |
| *SF3B1* | 0 (0.00%) | 0 (0.00%) | 0 (0.00%) |  |
| *CBL* | 5 (2.12%) | 4 (2.96%) | 1 (0.99%) | 0.396 |
| *PHF6* | 2 (0.85%) | 1 (0.74%) | 1 (0.99%) | 1.000 |
| *ZRSR2* | 0 (0.00%) | 0 (0.00%) | 0 (0.00%) |  |
| CR | 171 (72.46%) | 98 (72.59%) | 73 (72.28%) | 1.000 |
| Transplant | 38 (16.45%) | 17 (12.69%) | 21 (21.65%) | 0.102 |

WBC, white blood cell counts- HGB, hemoglobin- PLT, platelet- LDH, lactate dehydrogenase- BM, bone marrow- PB, peripheral blood- CR, complete remission. **Bold value indicates significant *P*-value.**

**Table S3 The catalog number of antibodies**

| Reagent | Company | Catalog number |
| --- | --- | --- |
| α-Tublin | ProteinTech, Wuhan, China | 66031-1-Ig |
| Cleaved-PARP | Cell Signaling Technology | 5625 |
| P53 | Cell Signaling Technology | 2527 |
| Cleaved caspase 3 | Cell Signaling Technology | 9664 |
| p27 Kip1 | Cell Signaling Technology | 3686 |
| p21 Waf1/Cip1 | Cell Signaling Technology | 2947 |
| Cyclin D1 | Cell Signaling Technology | 2978 |
| CDK4 | Cell Signaling Technology | 12790 |
| SLC25A21 | Affinity Biosciences LTD, Jiangsu, China | DF4172 |
| Cleaved caspase9 | Affinity Biosciences LTD, Jiangsu, China | AF5240 |
| BAX | ProteinTech, Wuhan, China | 505992-2-Ig |
| BCL2 | ProteinTech, Wuhan, China | 12789-1-AP |
| TOM20 | ProteinTech, Wuhan, China | 66777-1-Ig |
| Ki67 | Servicebio, Wuhan, China | GB111499 |
| HRP-labeled Goat Anti-Rabbit IgG(H+L) | Beyotime, Shanghai, China | A0208 |
| HRP-labeled Goat Anti-Mouse IgG(H+L) | Beyotime, Shanghai, China | A0216 |
| goat anti-rabbit IgG-HRP | Servicebio, Wuhan, China | G1213 |
| antibody Cy3 conjugated Goat Anti-Rabbit IgG (H+L) | Servicebio, Wuhan, China | GB21303 |
| Fluorescein (FITC)–conjugated Affinipure Goat Anti-Mouse IgG (H+L) | ProteinTech, Wuhan, China | SA00003-1 |
