# Supplementary figures and images for: SLC25A21 correlates with the prognosis of adult acute myeloid leukemia through inhibiting the growth of leukemia cells via downregulating CXCL8
Source: Cell Death Dis. 2024 Dec 20;15(12):921. doi: 10.1038/s41419-024-07308-y (PMC11662024; doi:10.1038/s41419-024-07308-y)

Figure 3 H.

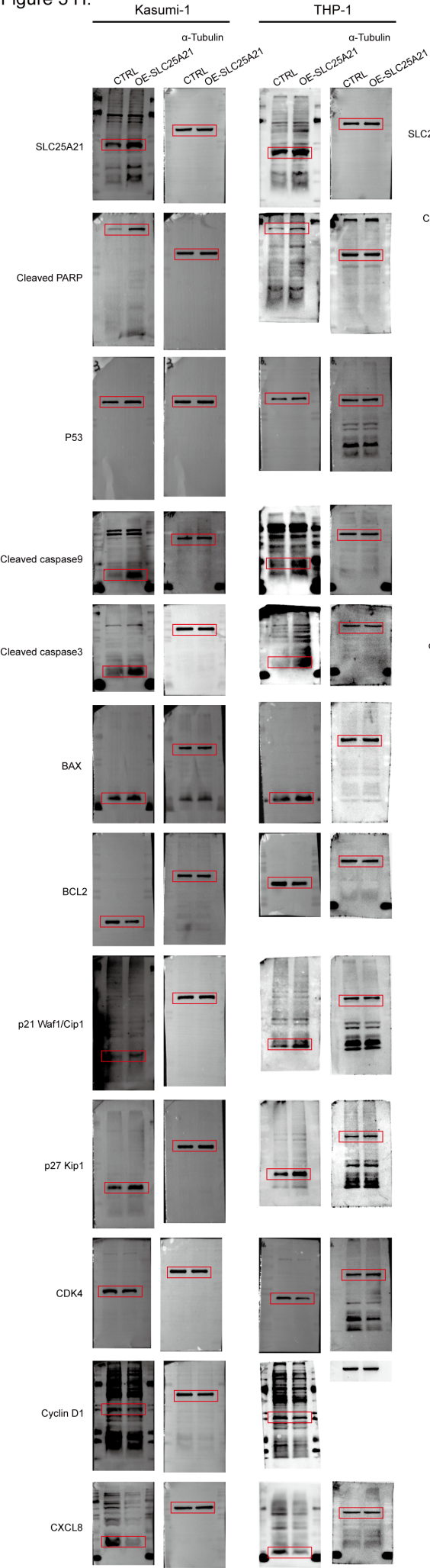

Figure 4. Tissue

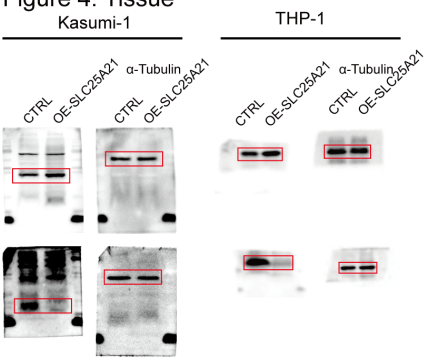

Figure 5. D

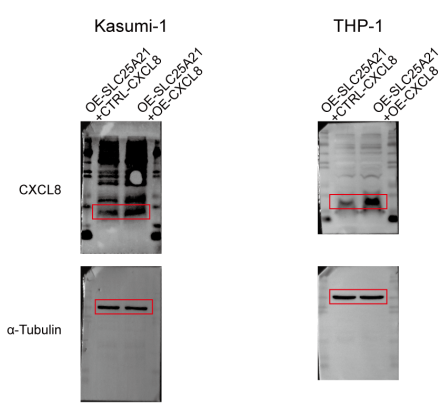

Supplement: Supplementary file 2 — Original WB data [file 41419_2024_7308_MOESM2_ESM.pdf]
